# Cylindrical 3D printed configurable ultrasonic lens for subwavelength focusing enhancement

**DOI:** 10.1038/s41598-020-77165-0

**Published:** 2020-12-15

**Authors:** Sergio Castiñeira-Ibáñez, Daniel Tarrazó-Serrano, Antonio Uris, Constanza Rubio, Oleg V. Minin, Igor V. Minin

**Affiliations:** 1 Centro de Tecnologías Físicas, Acústica, Materiales y Astrofísica, Universitat Politècnica de València, Camí de Vera s/n, 46022 Valencia, Spain; 2 Tomsk Polytechnic University, 36 Lenin Avenue, Tomsk, 634050 Russia

**Keywords:** Physics, Applied physics, Acoustics

## Abstract

In this study, we report the characteristics of acoustic jets obtained through a mesoscale (radius less than 5 wavelengths) ABS cylinder made with a 3D printer. We have analyzed the influence of cylinder size on the characteristic parameters of an acoustic jet, such as maximum acoustic intensity at focus, Full Width at Half Maximum and length of Acoustic Jet. FWHM below 0.5 wavelength in AJ was experimentally obtained. It has been observed that there are two operating regimes depending on the cylinder radius: the resonant and the non-resonant. In the resonant regime, the excitation of Whispering Gallery Modes results in optimal parameter values of the acoustic jet. However, as it is a resonant regime, any minimal variation in cylinder size, working frequency or refractive index would make resonance disappear. In non-resonant mode, a phononic crystal has been embedded inside the cylinder and the characteristic parameters of the acoustic jet have been studied. These have been observed to improve. Finally, we have shown that curved acoustic jets can be obtained with the ABS cylinder with a phononic crystal embedded inside.

## Introduction

Managing to focus energy into a region with smaller dimensions than the wavelength, that is, subwavelength focusing, has been one of the challenges of the scientific community. A physical phenomenon with which subwavelength focusing is achieved is the photonic nanojet (PNJ), which consists of a narrow electromagnetic beam that emerges from the shadow surface of a dielectric microsphere or micro cylinder. The PNJ was initially introduced by Chen et al.^1^ and, because a subwavelength beam waist and a high-intensity peak^2,3^ are obtained, it has attracted great interest in the scientific community. In addition, PNJs have applications in the design of different types of sensors and instrumentation^4–11^. The key parameters in the study of PNJs are light intensity, Full Width at Half Maximum (FWHM) and focal distance. It has been shown that a PNJ emerges from the shadow surface of a sphere or a dielectric cylinder with diameters of several wavelengths and with a refractive index with respect to the surrounding medium of less than two^3^. PNJs with dielectric cuboids have also been achieved^12,13^.

Since there is a formal analogy between electromagnetic waves and acoustic waves, the results obtained for electromagnetic waves can be transferred to acoustic waves, taking into account their intrinsic differences. In this sense, the existence of an intense acoustic field located on the shadow surface of a dielectric sphere was first demonstrated in simulations by Minin and Minin^14^. They called this phenomenon “acoustojet” (AJ). The AJ phenomenon was experimentally demonstrated using a Rexolite sphere with a radius of 4.14 wavelengths immersed in water and in the ultrasonic range at the frequency of 1.01 MHz. They obtained a shadow surface acoustic beam from the sphere with a 14.4 dB intensity gain and a FWHM of half a wavelength^15^. Similar experimental results were obtained for a cylindrical geometry^16^. It has also been established, both experimentally and by means of simulations, that a rectangular trapezoidal particle of Rexolite immersed in water and impacted by a plane ultrasonic wave can generate a curved acoustic beam with a radius of curvature substantially smaller than the wavelength in water^17^. On the other hand, AJ has been obtained experimentally using a hollow polyethylene tube filled with perfluorinated oil and immersed in water, olive oil and ethanol^18^. AJs have also been obtained using a hollow ABS sphere filled with a mixture of water and ethanol in different percentages. It was observed that, depending on the percentage of each liquid inside the ABS sphere, a subwavelength resolution could be achieved^19^. Recently, Leão-Neto et al.^20^ have reported numerically and experimentally that a subwavelength focus could be obtained by using a Rexolite sphere with another smaller carbon steel sphere inside, i.e. a core-shell shaped lens. Inspired by studies of photonic jets that improve optical properties using multilayer designs with different refractive indices, in this study we present an engineered two-layer cylinder in which we modify the refractive index of the inner layer by using a metamaterial, such as phononic crystal (PhC)^21^. There are artificial heterogeneous materials formed by scatterers that, when embedded in a medium with different elastic properties, have the ability to prevent certain frequency bands from propagating through them. The physical mechanism that explains this phenomenon is reduced to the scattering based on Bragg’s law. Both the arrangement of the scatterers in the network and their size are decisive in defining the attenuation bands in which there is no transmission of the sound wave. Furthermore, the density contrast between the scatterers and the host medium is also a determining factor, being necessary for the phenomenon to occur. In our case, the PhC is formed by water cylinders embedded in ABS. This is intended to improve the key parameters in an AJ, such as intensity enhancement, FWHM and the length of AJ, among others.

In this paper, we present an underwater solid ABS cylindrical acoustic lens built with a 3D printer. We have chosen ABS material as it is the most common for 3D printing. The key parameters of the lens could be improved by embedding a PhC in the medium of the cylinder. First, we show that there is a significant variation in the key AJ parameters as a function of cylinder diameter, suggesting a cylinder size with maximum acoustic intensity and lowest FWHM. Subsequently, we introduce the PhC and, by modifying its size and position within the cylinder, the variation of the sound intensity, FWHM and length of the AJ were observed. In this way, we show that we can adjust the key AJ parameters by modifying the size and position of the PhC. Finally, we show that by changing the angle of incidence, that is rotating the PhC, a curved acoustic jet can be obtained. The focusing capacity and key parameters of this cylindrical lens were obtained by using the Finite Element Method (FEM). In order to validate these numerical predictions, some experiments have been carried out using an automated robot in a water tank, reproducing FEM simulations as far as possible.

## Results

**Figure 1:**
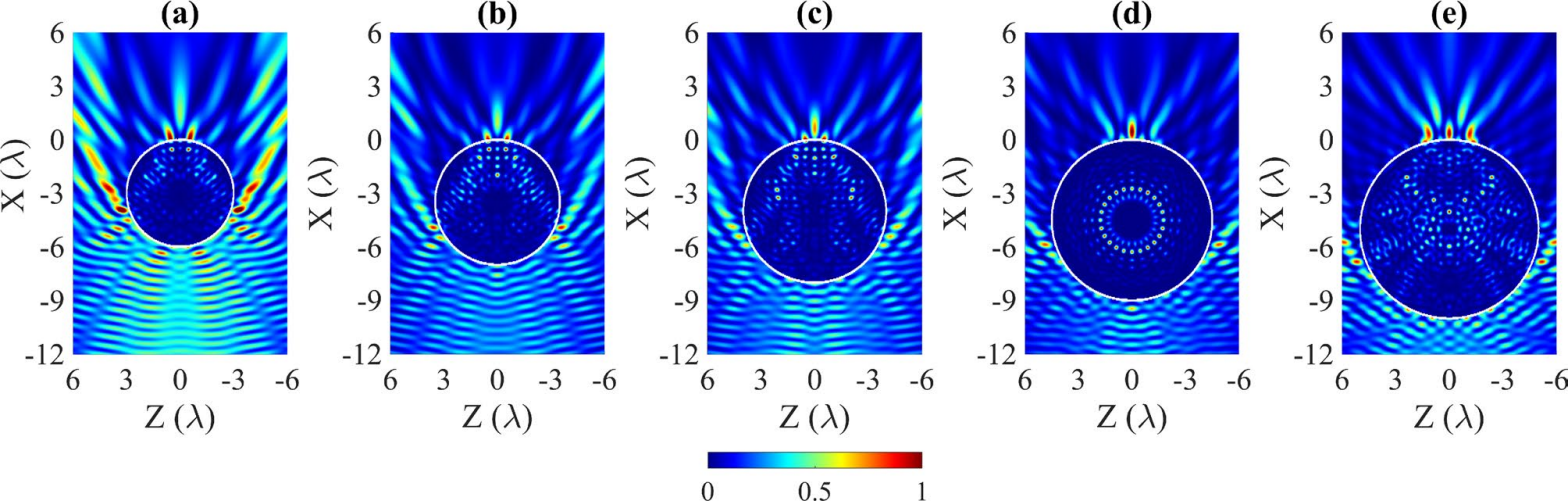
Normalized acoustic intensity planes ($I/I_{max}$) for different ABS cylinder sizes of (**a**) $3\lambda$, (**b**) $3.5\lambda$, (**c**) $4\lambda$, (**d**) $4.5\lambda$ and (**e**) $5\lambda$.

The effect of the size of the ABS cylinder on the parameters of the AJ was studied over a range of radius $R = \lambda -5\lambda$, where $\lambda$ is the host medium wavelength, which was water. The incident plane wave of $f=250\,\hbox { kHz}$ travels in the *X* direction and impinges. The normalized intensity $I/I_{max}$ distributions (where *I* and $I_{max}$ are the intensity at each point and the maximum intensity, respectively) in XZ planes around and inside the ABS cylinders with a radius *R* equal to 3$\lambda$-5$\lambda$, in steps of 0.5$\lambda$ is presented in Fig. 1a–e, respectively.

Table 1 shows $I_{max}$ ratio, FWHM, and the length of AJ for 3$\lambda$–5$\lambda$ ABS cylinder in steps of 0.5$\lambda$ . It can be seen that the $I_{max}$ ratio tends to increase in line with the radius of the cylinder until reaching a radius of 4.5$\lambda$ . From this point on, the maximum intensity decreases again. At the same time, the FWHM tends to decrease as the cylinder radius increases. The minimum FWHM value obtained was for the radius of 4.5$\lambda$ . From this value, the FWHM rises again. The same trend occurs with the length of the AJ. In our case, the length of the AJ is defined as the length through which the sound intensity decays 1/*e* to the peak value. As can be seen, the highest intensity values and the lowest FWHM values are obtained for the case of the cylinder with a radius of 4.5$\lambda$ . In this case, the Whispering Gallery Modes (WGM) inside the cylinder are excited, yielding a strongly localized sound pressure distribution with a symmetry pattern. We can see how there are two different operating regimes: the non-resonant and the resonant. In the non-resonant regime, the AJ is a consequence of the lensing effect and the radius of the cylinder determines the position of the AJ on the non-illuminated side of the cylinder, while in the resonant regime it is due to constructive interference. As seen in Fig. 1a–e, any detuning in the radius of the cylinder affects the resonance condition.

**Table 1:** Numerical parameter results for different ABS cylinder radii.

Radius ($\lambda$)	$I_{max}$ ratio	FWHM ($\lambda$)	Length of AJ ($\lambda$)
3.0	1.96	0.73	2.82
3.5	3.18	0.60	1.33
4.0	4.75	0.46	0.92
4.5	7.86	0.43	0.62
5.0	4.57	0.80	2.50

**Figure 2:**
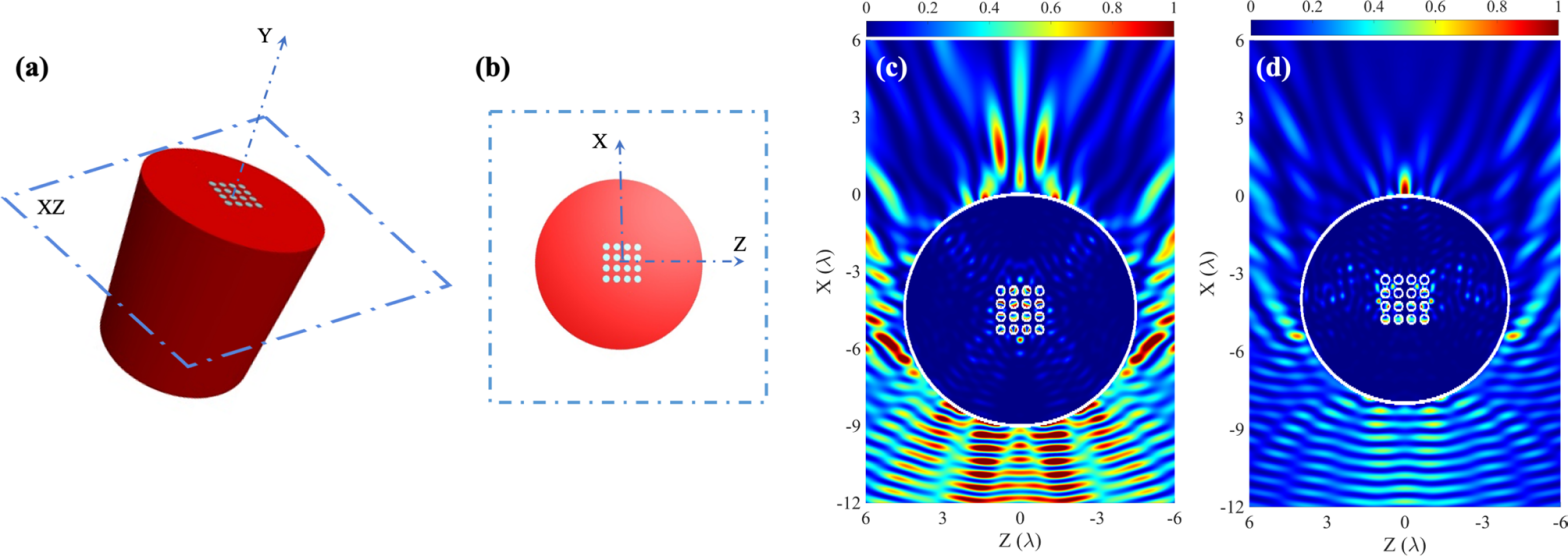
Schematic representation of the cylinder with a PhC embedded: (**a**) 3D view and (**b**) 2D view. Normalized intensity plane for numerical results for: (**c**) $4.5\lambda$ radius ABS cylinder with an embedded $4\times$ 4 PhC and (**d**) $4\lambda$ ABS cylinder with an embedded $4\times$ 4 PhC.

If we vary the effective refractive index of the cylinder, for example, by embedding a PhC inside the cylinder (Fig. 2a,b), the resonance condition also varies, as shown in Fig. 2c. Thus, one can choose an optimal radius of cylinder with a maximum intensity and a minimum FWHM of the AJ. In the non-resonant case the highest acoustic intensity and the lowest FWHM is obtained for a cylinder radius of 4$\lambda$. Let us now analyze the influence of varying the refractive index of a part of the cylinder on the characteristics of the AJ. The refractive index can be modified by varying the wave propagation speed in the medium. For this purpose, a PhC is embedded in the ABS cylinder. When it is included, it leads to a variation in the propagation speed of the ABS by an effective speed. This effective speed of sound ($c_{eff}$), is given by $c_{eff}=c_{host}/\sqrt{(1+ff)}\;$^22^. $c_{host}$ is the sound propagation speed in the host medium (ABS) where the PhC is embedded. *ff* is the ratio of the area occupied by the scatterers and the medium (ABS). In our case, a square lattice PhC with a scatterers’ radius (*r*) equal to 1 mm and a distance between them (*a*) equal to 3 mm was chosen. The influence of different sizes of the PhC grid was studied. PhC size is defined by the number of row column scatterers. The objective was to see which PhC size improves the key acoustic parameters ($I_{max}/I_0$, FWHM and AJ). Table 2 shows the numerical results for these sizes. Importantly, size $0 \times 0$ matches a cylinder without embedded PhC with a $R = 4\lambda$. Given the results, it can be affirmed that size $4 \times 4$ improves the acoustic parameters. Therefore, a new study is carried out to evaluate the influence of the position of the PhC in the ABS cylinder. Initially, the PhC was centered. The holes were filled with water. Figure 2d shows the sound pressure distributions in XZ planes around and inside the ABS cylinder of radius $R = 4\lambda$ and with $4 \times 4$ PhC embedded inside.

**Figure 3:**
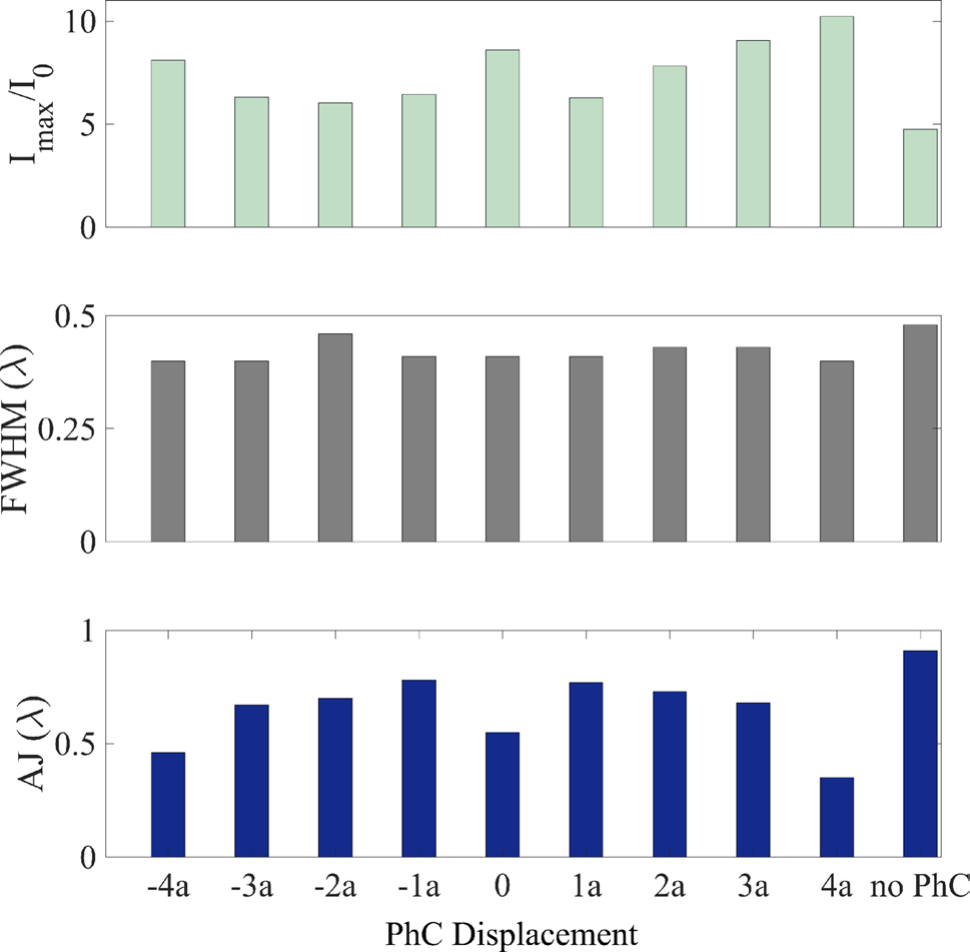
Acoustic parameter comparative for different PhC displacements inside the ABS cylinder.

This scenario is different from the previous one. It may be observed how there is sonic transmission through the PhC that produces a convergence of the sound beam, in addition to the lensing effect of the cylinder. The convergence of the wave front on the non-illuminated side is produced by the cylinder, that has a curved geometry, due to diffraction phenomenon. Furthermore, the wave travels changing propagation medium in such a way that pass from water to the cylinder and again to water. This change of material or medium redirects the wave front toward the cylinder. When these two effects overlap, the AJ is generated Fig. 1c. By introducing PhC, the diffraction that occurs due to geometry continues to exist. Instead, the insertion of the PhC causes the convergence of the wavefront, in such a way that the position of the focus moves and its intensity increases Fig. 2d. The sum of these two effects leads to an increase in the maximum intensity (more than that for a homogenous ABS cylinder with a radius of 4.5$\lambda$), and a decrease in the FWHM (less than that for a homogenous ABS cylinder with a radius of 4.5$\lambda$) and the length of the AJ, as can be seen in Table 2. This is caused by the increase in the focal power of the “droplet-lens”. By modifying the position of the PhC inside the cylinder, the convergence of the sound beam produced by the PhC will vary, as a consequence of which the AJ parameters will be affected. Figure 3 shows how the sound intensity, FWHM and length of the AJ vary when moving the PhC to the right (+ sign) or left (− sign). The displacements of the PhC multiples were multiples of the lattice constant *a*.

**Table 2:** Numerical parameter results for 4$\lambda$ radius ABS cylinder with different PhC sizes.

PhC (row $\times$ column)	$I_{max}$ ratio	FWHM ($\lambda$)	Length of AJ ($\lambda$)
$0\times 0$	4.75	0.46	0.92
$3\times 3$	7.00	0.42	0.83
$4\times 4$	8.61	0.41	0.55
$5\times 5$	8.27	0.41	0.37
$6\times 6$	6.66	0.45	0.23

**Figure 4:**
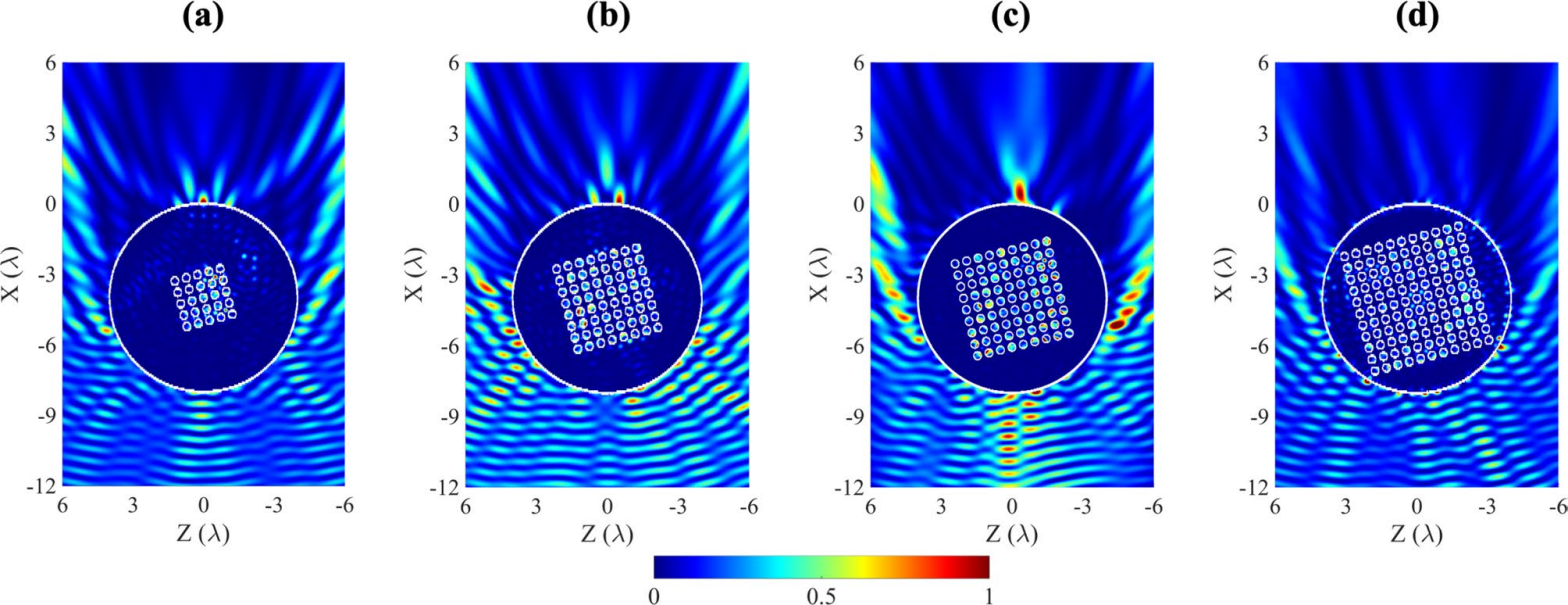
Normalized intensity plane ($I/I_{max}$) for ABS cylinder with an embedded (**a**) $5 \times 5$, (**b**) $8 \times 8$, (**c**) $9 \times 9$ and (**d**) $11 \times 11$ PhC which is rotated $\theta = 15^{\circ }$.

When the PhC is larger than $6 \times 6$, it may be observed that the sound field distribution changes when the PhC is rotated by an angle $\theta$. As the PhC grows in size, the wave attenuates, thus the focus behind the cylinder disappear. This effect is observed when the PhC is symmetric with respect to the wave incidence. By slightly rotating the cylinder, and therefore the PhC, the focus reappeared as an acoustic hook. The reason of this different behaviour is due to the rotation of the sample. When this occurs, the wave propagation direction does not coincide with the main direction of the PhC, and thus the attenuation obtained decreases. The asymmetry that has been introduced to the sample has again favoured the observed phenomenon. Figure 4 represents the sound pressure distributions in XZ planes around and inside the ABS cylinder of radius $R = 4\lambda$, $5\times 5$ (5 rows and 5 columns), $8\times 8$, $9\times 9$ and $11\times 11$ PhC when an angle $\theta = 15^{\circ }\,$ has been rotated. It may be observed that the shape of the AJ is curved. This effect is known as an acoustic hook^17^. This phenomenon is caused by broken symmetry. A video can be seen in the Supplementary Information S1, varying $\theta$ from $0^{\circ }\,$ to $45^{\circ }\,$ in the $9 \times 9$ case.

**Figure 5:**
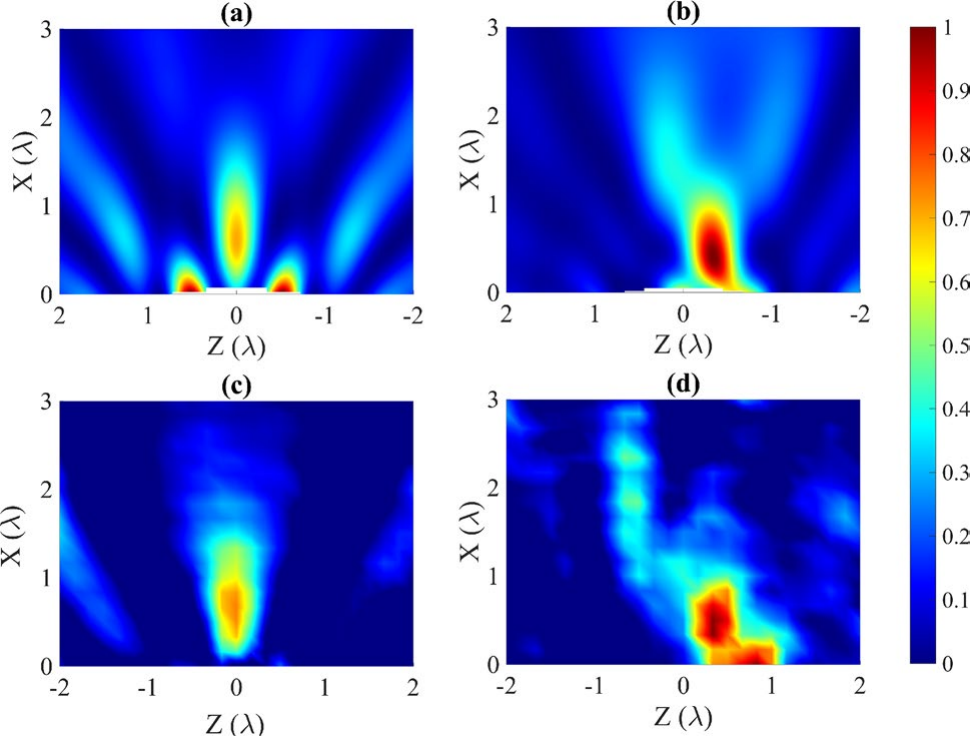
Normalized intensity planes ($I/I_{max}$) for $R=4\lambda$ ABS cylinders. (**a**,**b**) correspond to numerical solutions with and without embedded $9 \times 9$ PhC rotated $\theta = 15^{\circ }\,$, respectively. (**c**,**d**) correspond to experimental measurements for same cases stated before.

**Figure 6:**
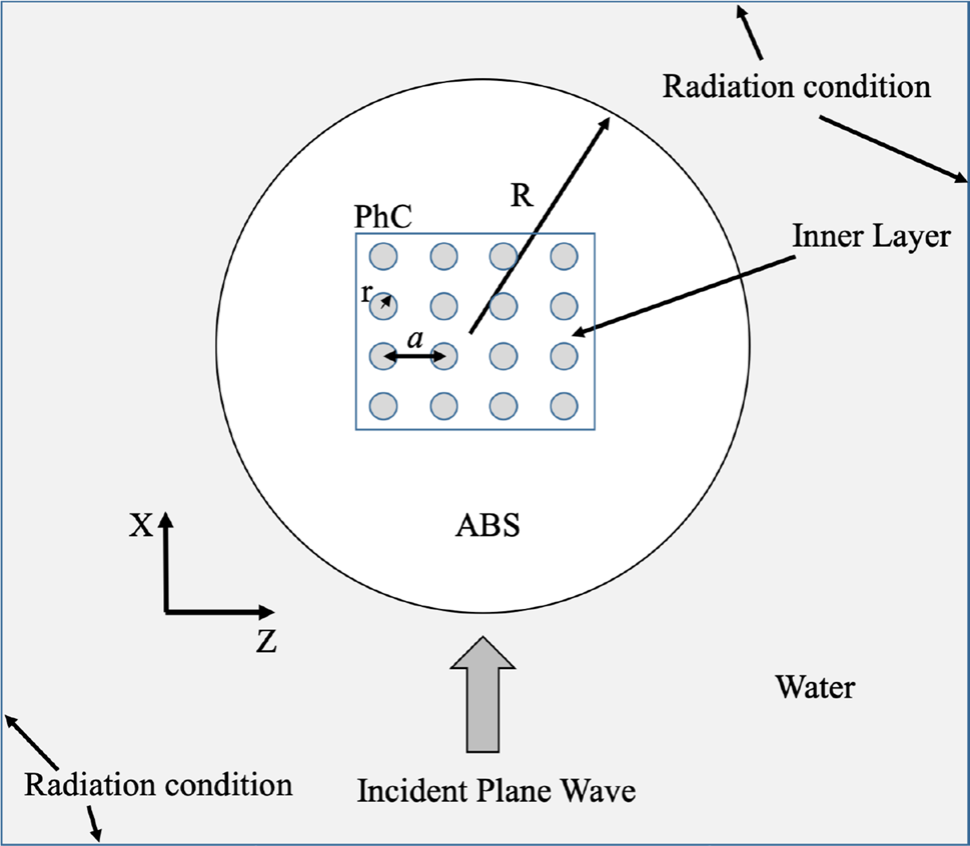
Scheme of the geometry and boundary conditions of the implemented FEM model.

The ultrasonic immersion technique was used to experimentally verify the Finite Element simulation results (see Fig. 6; “Methods” section). An ABS cylinder of radius $R = 4\lambda$ and an ABS cylinder of radius $R = 4\lambda$ with a 9-row and 9-column ($9 \times 9$) PhC embedded inside were measured. Figure 5a,b shows numerical results for ABS cylinder with and without the embedded $9 \times 9$ PhC, respectively. Figure 5c,d shows the experimental results in the same way as stated before. In this way, it can be verified that, numerically, the experimental results are as expected. The AJ value for the experimental case of the ABS cylinder without embedded PhC is 1.1$\lambda$. Comparing this with the numerical AJ value (0.96$\lambda$ ), it can be stated that FEM models are consistent with the experimental results. On the other hand, the side lobes cannot be seen because the hydrophone could not be compromised measuring an area so close to the ABS cylinder. A slight blow could seriously damage the equipment. Figure 5d clearly shows the AJ bending effect. The emission of the transducer is not really plane wave, although the particles were at a relatively long distance from the transducer. However, the experimental and simulated results agree closely , which allows us to validate the results of the simulations presented in this study.

## Discussion

In this study, we have presented an ABS cylinder made with a 3D printer that achieves AJ with a FWHM below $\lambda /2$. Firstly, the influence of the cylinder size has been analyzed. It has been observed that there are two different operating regimes depending on the cylinder radius: the non-resonant and the resonant. The highest intensity values and the lowest FWHM values are obtained in the resonant regime, giving rise to the WGM inside the cylinder. However, a slight variation in the size of the cylinder, the frequency of the incident wave or the refractive index of the material would make the WGM disappear. In the non-resonant regime, it has been observed that there is an optimal radius of cylinder that gives rise to an AJ with a maximum intensity and a minimum FWHM, which in our case is the cylinder with a radius of 4$\lambda$. Using this cylinder as a base, a PhC has been embedded in it with which it is possible to increase the intensity of the AJ, reduce the FWHM and decrease the length of the AJ. Also, by varying the position of the PhC inside the cylinder, the parameters of the AJ vary. This is due to the sum of two effects: the lensing effect of the cylinder and the convergence of the sound beam due to the sonic transmission through the PhC.

It has been observed that when we embed a PhC larger than $6 \times 6$, the distribution of the sound field changes when the PhC is rotated by an angle $\theta$, giving rise to a curved AJ. This effect is known as an acoustic hook and is due to the symmetry breaking. Furthermore, we have demonstrated that it is possible to construct, in a fast and versatile way, by means of 3D printers, ultrasonic lenses with adjustable parameters at any time. The design has been demonstrated by FEM simulations and validated by experiments. In our opinion, these results may be used for designing new ultrasonic devices. Moreover, from increasing the acoustic intensity level and increasing spatial resolution, these devices could be used in new designs of medical instrumentation for drugs manipulation. In engineering field, our device could be used in particle or object trapping.

## Methods

### FEM models used in numerical simulations

The numerical results were obtained using the so-called Finite Elements Method software COMSOL Multiphysics. To reduce the computational cost and take advantage of the boundary and initial conditions of the problem, a two-dimensional model has been used. The problem was solved by considering the solid-structure coupling as being able to obtain the solution in which the ABS cylinders had two propagation sound speeds (longitudinal and shear). Therefore, two perfectly-coupled physical modules were used to obtain the solution. An acoustic module contained the solution for the host domain. It was defined as water with typical sound speed ($c=1500\,\hbox { m/s}$) and density ($\rho = 1000\,\hbox { kg/m}^3$) values. The background pressure field was selected to emulate the plane-wave incident. To emulate an infinite medium and avoid reflections, the edges of the host were defined as radiation contours. In this sense, the Sommerfeld condition is achieved. The acoustic-structure module solved the acoustic-structure interaction in the cylinder. Cylinder probes were implemented as isotropic linear elastic materials using three ABS physical parameters: longitudinal wave speed ($c_l=2250\,\hbox { m/s}$), shear wave speed ($c_s=1025\,\hbox { m/s}$) and density ($\rho _{ABS}=1050\,\hbox { kg/m}^3$). To achieve a perfectly-coupled system, cylinder contours, as well as PhC contours inside the cylinder, are included in the multi-physics module so that solid-structure interaction is achieved. The mesh was defined as a triangular typology. In order to avoid numerical dispersion, the maximum element size was $\lambda$/10. Figure 6 shows the details of the implemented FEM model.

### Experimental set-up

**Figure 7:**
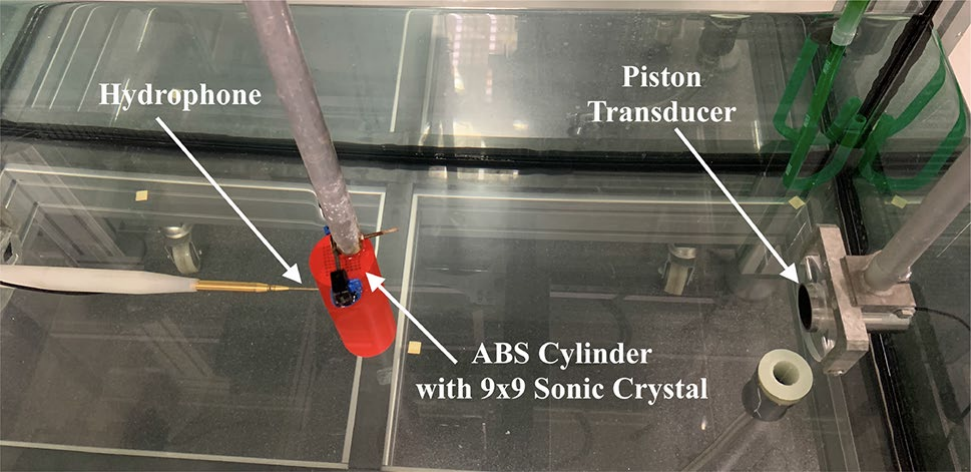
Photography with the detail of the different parts of the experimental set-up.

Experimental measurements were taken using an automated high-precision system. This system performs high-precision measurements using a step-by-step robot-driven hydrophone in a water tank. This polyvinylidene fluoride needle hydrophone is a model from Precision Acoustics, with a diameter of 1.5 mm. Furthermore, it has a flat answer (± 4 dB) between 200 kHz and 15 MHz. The experimental system consists of a piston-type transducer that serves as an emitter. This Imasonic-signed emitter has a 250 kHz working frequency and 32 mm of active diameter. A Picoscope Model 3224 digital oscilloscope has been used to digitize and amplify the measurements. The steps between each measurement point are 1 mm. This step size scanning allows a resolution of $\lambda$/6 to be obtained in experimental measurements. The measuring tank has dimensions of 0.5 $\times$ 0.5 $\times$ 1 m$^3$. The sample is centred in the water tank. Therefore, 0.25 m is separated from each side of the tank, representing a first reflection time of approximately 200 $\upmu$s. Due to our software allows time screening selection, the first reflections can be filtered (the digital oscilloscope sampling allows 1 $\upmu$s steps selection). However, the edge effect of the cylinder was considered, and it is reflected in the measurement plane. Wherewith, in the model we considered free field due to in the experiments we are filtering the reflections and considering only the direct field. To validate the results of the simulations, two ABS cylinders were printed. A solid ABS cylinder. of radius $R = 24\,\hbox { mm}$ ($4\lambda$), and an ABS cylinder, of radius $R = 24\,\hbox { mm}$ ($4\lambda$) with an embedded PhC. The PhC was square lattice with circular holes of radius $r = 1\,\hbox {mm}$ and lattice constant $a = 3\,\hbox { mm}$. The PhC was centred and had 9 rows and 9 columns ($9 \times 9$). Figure 7 shows the experimental set-up with one of the mounted probes.

## Supplementary information

Supplementary information.
