# Supplementary figures and images for: Cylindrical 3D printed configurable ultrasonic lens for subwavelength focusing enhancement
Source: Sci Rep. 2020 Dec 15;10:20279. doi: 10.1038/s41598-020-77165-0 (PMC7738512; doi:10.1038/s41598-020-77165-0)

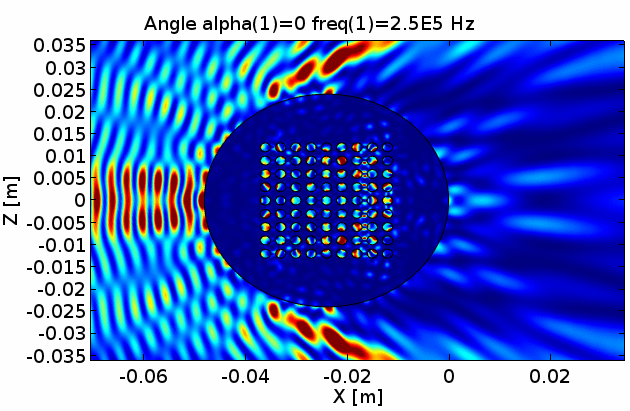

Supplement: Supplementary file 1 — Supplementary information. [file 41598_2020_77165_MOESM1_ESM.gif]
